# Evaluating Photodynamic Therapy vs. Subthreshold Micropulse Laser for Central Serous Chorioretinopathy: A Retrospective Study

**DOI:** 10.1055/a-2542-4969

**Published:** 2025-04-16

**Authors:** Tahm Spitznagel, Katrin Fasler, Jay Zoellin, Jeanne Martine Gunzinger, Chiara Sommer, Stephan Kinzl, Daniel Rudolf Muth, Ferhat Turgut, Amr Saad, Matthias Becker, Gabor Mark Somfai, Sandrine Zweifel

**Affiliations:** 1 Department of Ophthalmology, Stadtspital Zürich Triemli, Zurich, Switzerland; 2 Spross Research Institute, Zurich, Switzerland; 3 Department of Ophthalmology, University Hospital Zurich, Switzerland; 4 Department of Ophthalmology, Hunter New England Health, New Lambton, Australia; 5 Department of Clinical Neuroscience, Division of Eye and Vision, Karolinska Institiutet, Stockholm, Sweden; 6 Augenklinik, Gutblick Research, Pfäffikon, Switzerland; 7 Department of Ophthalmology, Semmelweis University, Budapest, Hungary; 8 Department of Ophthalmology, Heidelberg University, Heidelberg, Germany

**Keywords:** CSCR, chorioretinitis centralis serosa, photodynamic therapy, subthreshold micropulse laser, Chorioretinitis centralis serosa, photodynamische Therapie, Subthreshold-Micropulse-Lasertherapie, CRCS

## Abstract

**Background** Our study aims to compare the efficacy of half-dose photodynamic therapy with verteporfin (PDT) and subthreshold micropulse laser (SML) in patients with central serous chorioretinopathy (CSCR) with regards to subretinal fluid (SRF) resorption, visual acuity (VA), and central subfield thickness (CST).

**Patients and Methods** We conducted a retrospective multicentre clinical study at the Departments of Ophthalmology at the University Hospital Zurich and Stadtspital Zürich, Switzerland. The study included patients with acute and chronic CSCR who underwent PDT, SML, or both sequentially between June 1, 2020 and December 31, 2023. The primary outcome was the reduction in SRF at three and six months post-treatment. The secondary outcomes included change in CST VA at the same intervals.

**Results** A total of eighty-one eyes were analysed (33 PDT, 35 SML, thirteen combined). SRF reduction was statistically significant at both three and six months in the PDT (p < 0.001) and SML groups (p < 0.001). The combined treatment group showed a significant reduction in SRF only at six months (p = 0.001). At three months, PDT resulted in a significantly greater SRF reduction than the combined group.

**Conclusions** Both PDT and SML demonstrated improvements in SRF, CST and VA, with SML presenting as a comparable alternative, particularly in cases where access to verteporfin is limited.

## Zusammenfassung

**Zweck** Ziel unserer Studie ist der Vergleich der Wirksamkeit der halbdosierten photodynamischen Therapie mit Verteporfin (PDT) und der Subthreshold-Micropulse-Lasertherapie (SML) bei Patienten mit Chorioretinitis centralis serosa (CSCR) hinsichtlich der Resorption subretinaler Flüssigkeit (SRF), des Visus (VA) und der zentralen Subfelddicke (CST).

**Patienten und Methoden** Eine retrospektive multizentrische klinische Studie wurde an den Augenkliniken des Universitätsspitals Zürich und des Stadtspitals Zürich, Schweiz, durchgeführt. Die Studie umfasste Patienten mit akuter und chronischer CSCR, die sich zwischen dem 1. Juni 2020 und dem 31. Dezember 2020 einer PDT, SML oder beiden Therapien unterzogen haben. Der primäre Endunkt war die Reduktion der SRF 3 und 6 Monate nach der Behandlung. Zu den sekundären Ergebnissen gehörte die Veränderung der CST und der VA zu denselben Zeitpunkten.

**Ergebnisse** Insgesamt wurden 81 Augen analysiert (33 PDT, 35 SML, 13 mit beidem). Die SRF war sowohl nach 3 als auch nach 6 Monaten in der PDT- (p < 0,001) und in der SML-Gruppe (p < 0,001) statistisch signifikant reduziert. Die kombinierte Behandlungsgruppe zeigte eine signifikante Verringerung der SRF nur nach 6 Monaten (p = 0.001). Nach 3 Monaten führte die PDT zu einer signifikant stärkeren Verringerung der SRF als die kombinierte Gruppe.

**Konklusion** Sowohl PDT als auch SML zeigten Verbesserungen der SRF, der CST und der VA, wobei SML insbesondere in Fällen mit begrenztem Zugang zu Verteporfin eine vergleichbare Alternative darstellt.

## Background

Central serous chorioretinopathy (CSCR) is a complex choroidal/retinal disorder primarily characterised by an accumulation of subretinal fluid (SRF) and serous retinal detachments (SRD) typically overlying areas of altered choroidal structure (such as thickened or hyperpermeable vessels and attenuated choriocapillary layer) 1 , 2 , 3 . The most widely accepted pathophysiological model suggests primary choroidal dysfunction influenced by venous congestion, inflammation, steroid metabolism anomalies, and genetic predispositions 2 , 3 . This dysfunction leads to SRF accumulation at leakage points arising from micro-tears in the retinal pigment epithelium (RPE) 4 . Acute cases of CSCR (< 6 months) are characterised by SRF accumulation that spontansously resolves, whereas chronic cases (> 6 months) are associated with longstanding extensive serous detachments and atrophic changes in the outer retina/RPE 4 , 5 . Recurrent and/or chronic cases leading to RPE atrophy may result in significant visual loss; this predominantly affects middle-aged male patients 6 , 7 .

Treatment options for CSCR are limited. Initially, a watchful waiting approach is recommended for up to six months, after which half-dose or half-fluence PDT is generally the preferred treatment. This approach is supported by evidence from randomised controlled trials including the VICI, PLACE, and SPECTRA studies 7 , 8 , 9 , 10 , 11 .

Due to the intermittent scarcity of PDT primarily caused by limited availability of verteporfin (Visudyne), subthreshold micropulse laser (SML) therapy has emerged as the main alternative for treating CSCR. However, evidence supporting the efficacy of SML remains limited. Several retrospective studies have explored SML with varying wavelengths (810 nm, 577 nm, 532 nm, and 527 nm) and distinct treatment protocols reporting cases of SRF resolution in CSCR between 36% and 100%. Some of these studies also suggest potential for accelerated SRF resolution in acute CSCR. Nevertheless, the only large randomised prospective trial (PLACE) has yielded findings that suggest SML to be less effective than photodynamic therapy (PDT) in the treatment of chronic CSCR 9 , 12 , 13 , 14 , 15 , 16 , 17 , 18 .

The purpose of this study was to compare the efficacy of SML (577 nm wavelength) and PDT in both acute and chronic CSCR focusing on SRF resolution and improvement in visual acuity (VA).

## Patients and Methods

### Ethics

Institutional review board approval for both centres (University Hospital Zürich, BASEC-No. PB_2016 – 00 264; Stadtspital Zürich 2023 – 00 901) was obtained from the Cantonal Ethics Committee of Zurich. All patients provided informed consent for their clinical data to be published. The study strictly adhered to the tenets of the Declaration of Helsinki.

### Study design, participants, data collection

This is a retrospective clinical study based on a review of the patient medical records, including data from multimodal imaging. Inclusion criteria were patients ≥ 18 years of age with acute or chronic CSCR who had received either PDT, SML or both treatments between June 2015 and June 2023 with a minimum follow-up of three months. CSCR was confirmed through clinical examination and imaging. All patients underwent at least one OCT scan (Spectralis, Heidelberg Engineering, Heidelberg, Germany), while many additionally received autofluorescence and/or fluorescein angiography (FA)/indocyanine green angiography (ICG) using the same device except for those with allergies or contraindications. Chronic CSCR was defined as the presence of subretinal fluid persisting for more than six months along with imaging features such as RPE atrophy, diffuse RPE decompensation, or widespread serous retinal detachments observed on OCT, FA, or ICG, and patient-reported symptoms of prolonged visual disturbances or documented history of recurrent CSCR episodes. Exclusion criteria were secondary choroidal neovascularisation and concurrent intravitreal therapy. However, patients with previous PDT treatment (> 2 years prior) or current/past local or systemic steroid therapy were eligible for inclusion. The baseline visit was defined as the date of their first PDT or SML treatment. Some patients received a second, different treatment within six months before baseline or during the six-month follow-up. If the second therapy was administered before the three-month follow-up, data from both three and six-month follow-up were included in the “combined” treatment group; if the second treatment was administered between three and six-month follow-up, only the six-month data was included in the combined treatment group.

The following parameters were extracted from the patient records: demographic data, corrected VA on a decimal Snellen scale (converted to logMAR for statistical analysis), duration of SRF prior to treatment, current and past steroid treatments, and indications for a second SML or PDT treatment. Parameters extracted from OCT images included maximum SRF height (measured perpendicularly to the RPE at the point of greatest vertical extent using the calliper function in Heidelberg HEYEX), foveal involvement of SRF, and central subfield thickness (CST). The primary endpoint was SRF reduction at three and six-months post-SML or PDT treatment, while secondary endpoints included changes in CST and VA over the same intervals. The methodology largely mirrors that of our previous paper on the efficacy of SML, with some adaptations specific to this analysis 18 .

### PDT treatment

Subjects were assigned to receive either half-dose (patients at Stadtspital Zurich) or half-fluence (patients at University Hospital Zurich) PDT treatment. Verteporfin was administered intravenously at a reduced dose followed by activation with a diode laser at 689 nm applied to the affected area of the retina. After the intervention, patients were instructed to wear sunglasses and minimise exposure to direct sunlight (including protective clothing and avoid outdoor activities) for forty-eight hours due to the risk of potential photosensitivity reactions. Each patient was also provided with a wristband containing information about their recent laser treatment, which they were instructed to wear for one week.

### SML treatment

Subjects received SML treatment following Chhablaniʼs established protocol 19 . Confluent 577 nm laser spots were applied, each measuring 100 µm in diameter with a 100 ms pulse and a 5% duty cycle. Laser power was then titrated at the arcades until a minimally perceptible laser burn was achieved with the definitive treatment power set at 30% of the total titrated energy 18 . The study encompassed two subgroups based on the type of treatment guidance used: (1) FA/ICG-guided laser, whereby the treatment area encompassed leakage spots on FA and areas of choroidal hyperpermeabilities on ICG; (2) OCT-guided laser. In cases where FA/ICG imaging could not be performed due to patient-specific factors such as allergies, comorbidities, or personal preference, treatment guidance was based solely on OCT imaging. Patients in this subgroup received OCT-guided therapy, focusing on the total area of SRF as delineated on OCT.

### Statistical analysis

Descriptive statistics were used to summarise demographic data and baseline values for SRF, CST, and VA. Data distributions were presented as means, medians, interquartile ranges (IQRs), and standard deviations (SDs). For inferential statistics, linear mixed-effects models (LMMs) were used, all of which were fitted using restricted maximum likelihood (REML) estimation. Dependent variables included SRF, CST, VA, and normalised measures of SRF and CST, expressed as percentages relative to baseline for each timepoint.

Within each treatment group, fixed effects included Timepoint, Duration of SRF, Treatment Plan, and CSCR Type with a random intercept for each patient. An interaction term between Timepoint and Treatment Group was introduced to compare across treatment groups. Hypothesis testing for fixed effects was conducted using p-values estimated by the Satterthwaite method. Model fit was evaluated using Akaike Information Criterion (AIC) and Bayesian Information Criterion (BIC) values to ensure the best-fitting models. Post-hoc pairwise comparisons were performed using estimated marginal means (emmeans) with Kenward-Roger degrees of freedom adjustment, and p-values were corrected for multiple comparisons using Tukeyʼs method.

Additional analyses comparing SRF, CST, SRF percentage, CST percentage, and VA across treatment groups were conducted using either ANOVA or Kruskal-Wallis tests depending on data normality, which was assessed using the Shapiro-Wilk test. For Kruskal-Wallis tests, post-hoc pairwise comparisons were conducted using Dunnʼs test with Benjamini-Hochberg corrections, while Tukeyʼs HSD test was employed for ANOVA.

Data were visualised using boxplots with individual data points superimposed as scatter plots and patient trajectories represented by fine lines. Subgroup analyses were performed by extracting estimates and p-values from fixed effects in the LMMs to determine whether variables such as the duration of SRF before treatment, CSCR type (acute vs. chronic), and treatment modality (FA-guided therapy vs. OCT-guided therapy) acted as significant predictors of outcomes.

Statistical analyses were conducted using R software (version 4.1.3; R Foundation for Statistical Computing, Vienna, Austria, 2016). The threshold for statistical significance was set at an alpha of 0.05.

## Results

### Demographics and baseline characteristics

A total of eighty-one eyes from seventy-seven patients were included in the study; 33 eyes were treated with PDT, 35 eyes with SML, and 13 received combined treatment ( Table 1 ). 26/33 (78.79%) of the PDT group, 18/35 (51.43%) of the SML group and 11/13 (84.62%) of patients treated with both, showed signs of chronic CSCR. The median age was 51 in the PDT group, 48 in the SML group and 48 in the group “combined”. Men were predominantly represented at 85 – 93% in all groups. The baseline median value for the maximum SRF height was 158 µm (IQR 98 – 224 µm) in PDT patients, 139 µm (IQR 99 – 188.5 µm) in SML patients and 130 µm (101 – 166 µm) in patients treated with PDT and SML. The median VA (logMAR) was 0.2 (0.1 – 0.4) in PDT patients, 0.2 (IQR: 0.0 – 0.4) in SML patients and 0.2 (0.1 – 0.4) in patients treated with PDT and SML ( Tables 2 , 3 and 4 ).

**Table:** **Table 1** Baseline Characteristics.

Variable	N	PDT	SML	Combined	P value
Eyes	81	33	35	13	
Right eyes (%)	44	16 (48.48)	19 (54.29)	9 (69.23)	0.502
Left eyes (%)	37	17 (51.51)	16 (45.71)	4 (30.77)	0.502
Age median (IQR)		51 (45 – 56)	48 (41 – 58.5)	48 (43 – 55)	0.542
Gender					
Male (%)	71	29 (87.88%)	30 (85.71%)	12 (92.31%)	1.0
Female (%)	10	4 (12.12%)	5 (14.29%)	1 (7.69%)	1.0
Visual acuity in logMAR (IQR)		0.2 (0.1 – 0.4)	0.2 (0.0 – 0.4)	0.2 (0.1 – 0.4)	0.857
OCT characteristics					
Duration of SRF median in weeks (IQR)		37.07 (18.54 – 89.04)	23.71 (12.50 – 45.71)	65.29 (30.00 – 86.29)	0.156
Maximal SRF median in µm (IQR)		158 (98 – 224)	139 (99 – 188.5)	130 (101 – 166)	0.554
CST median in µm (IQR)		376 (332 – 449)	318 (284.5 – 384.5)	362 (279 – 417)	0.075
Presence of subfoveal SRF (%)		29 (87.88)	30 (85.71)	13 (100)	0.51
Corticosteroid therapy					
Prior corticosteroid therapy (%)	22	13 (39.39)	8 (22.86)	1 (7.69)	0.087
No prior corticosteroid therapy (%)	34	14 (42.42)	13 (37.14)	7 (53.85)	0.600
Unknown (%)	25	6 (18.18)	14 (40.00)	5 (38.46)	0.109

**Table:** **Table 2** Progression of functional and morphological parameters at baseline and at three and six months after PDT.

Variable	Baseline	3 months	6 months
n	33	33	23
VA in logMAR (IQR)	0.2 (0.1 – 0.4)	0.1 (0.0 – 0.3)	0.1 (0.0 – 0.2)
Maximal SRF median in µm (IQR)	158 (98 – 224)	0 (0 – 98)	85 (21.5 – 128.5)
CST median in µm (IQR)	376 (332 – 449)	280 (254 – 312)	289 (254.5 – 321.5)
Presence of subfoveal SRF (%)	29 (87.88)	8 (24.24)	10 (43.48)

**Table:** **Table 3** Progression of functional and morphological parameters at baseline and at three and six months after SML.

Variable	Baseline	3 months	6 months
n	35	35	30
VA in logMAR (IQR)	0.2 (0.0 – 0.4)	0.1 (0.0 – 0.375)	0.15 (0.0 – 0.3)
Maximal SRF median in µm (IQR)	139 (99 – 188.5)	70 (12 – 124.5)	67 (26 – 99.75)
CST median in µm (IQR)	318 (284.5 – 384.5)	275 (239.5 – 316.5)	274.5 (229 – 311.25)
Presence of subfoveal SRF (%)	30 (85.71)	18 (51.43)	18 (60.00)

**Table:** **Table 4** Progression of functional and morphological parameters at baseline and at three and six months after patients received both treatments.

Variable	Baseline	3 months	6 months
n	13	11	11
VA in logMAR (IQR)	0.2 (0.1 – 0.4)	0.1 (0.0 – 0.2)	0.0 (0.0 – 0.1)
Maximal SRF median in µm (IQR)	130 (101 – 166)	144 (47.5 – 196)	29 (0 – 90)
CST median in µm (IQR)	362 (279 – 417)	308 (248 – 396)	255 (244.5 – 264)
Presence of subfoveal SRF (%)	13 (100)	7 (63.64)	4 (36.36)

### Primary Outcomes

**Fig. 1:**
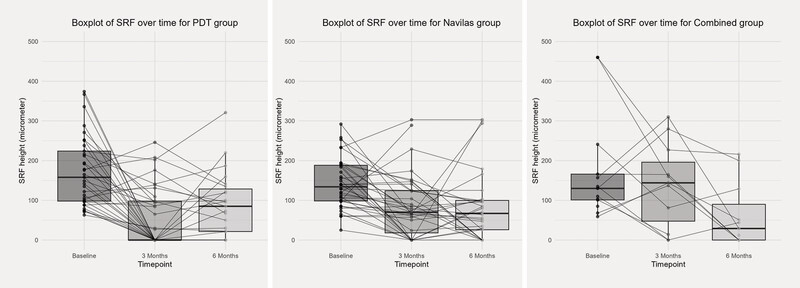
Boxplots of subretinal fluid (SRF) over time from baseline to three and six months.

The reduction in maximal SRF height was statistically significant at both three and six months in the PDT (p < 0.001) and SML (p < 0.001) groups. In the “combined” group, the reduction in maximal SRF height was significant only at six months (p = 0.001) ( Fig. 1 ).

**Fig. 2:**
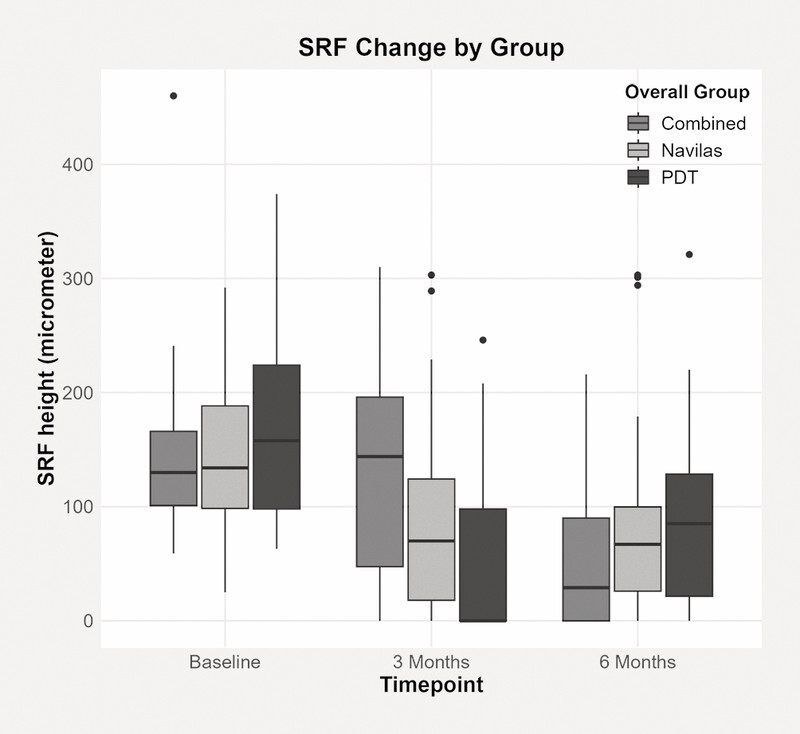
SRF change by group.

Between groups, maximal SRF height was only significantly different at three months post-treatment (p < 0.05), and this was observed only in the comparison between the PDT group and the “combined” group ( Fig. 2 ). There were no statistically significant differences between the “combined” and SML groups or between the PDT and SML groups.

### Secondary Outcomes

**Fig. 3:**
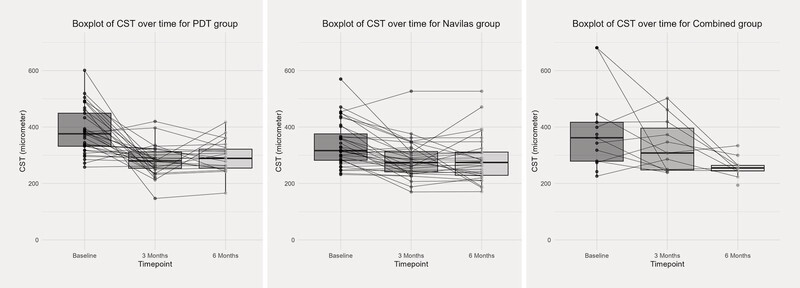
Boxplots of central subfield thickness (CST) over time from baseline to three and six months.

The reduction in CST was statistically significant at both three and six months in the PDT (p < 0.001) and SML (p < 0.001) groups ( Fig. 3 ). In the “combined” group, a significant reduction in CST was only observed at six months (p = 0.001).

**Fig. 4:**
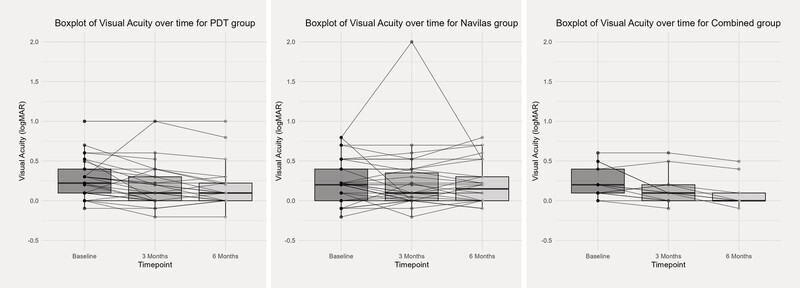
Boxplots of central visual acuity (VA) over time from baseline to three and six months.

Improvement in VA was statistically significant at both three and six months in the PDT (p = 0.001) and “combined” groups (p < 0.01), while no significant change in VA was found at either three or six months in the SML group ( Fig. 4 ).

**Fig. 5:**
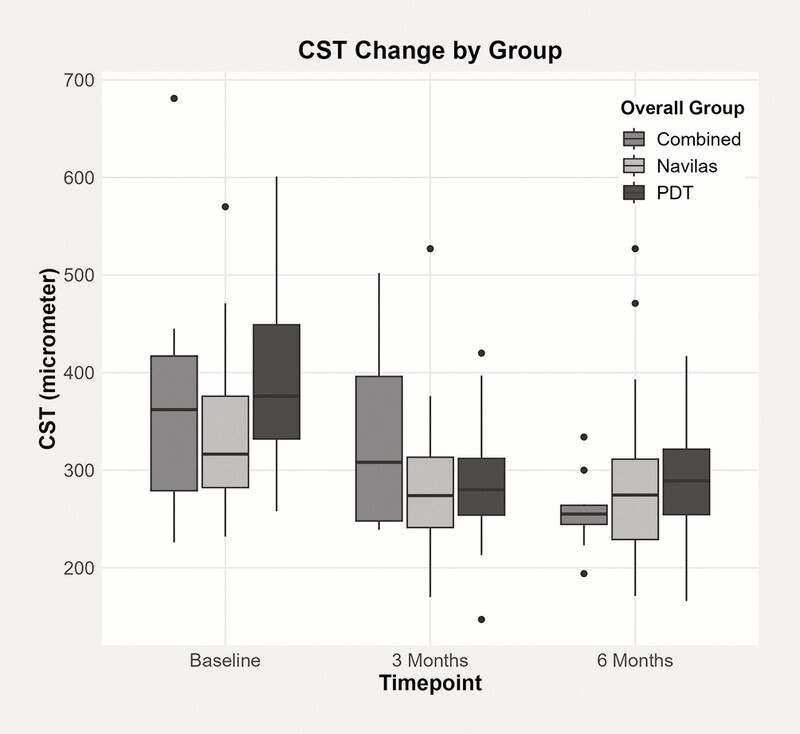
CST change by group.

**Fig. 6:**
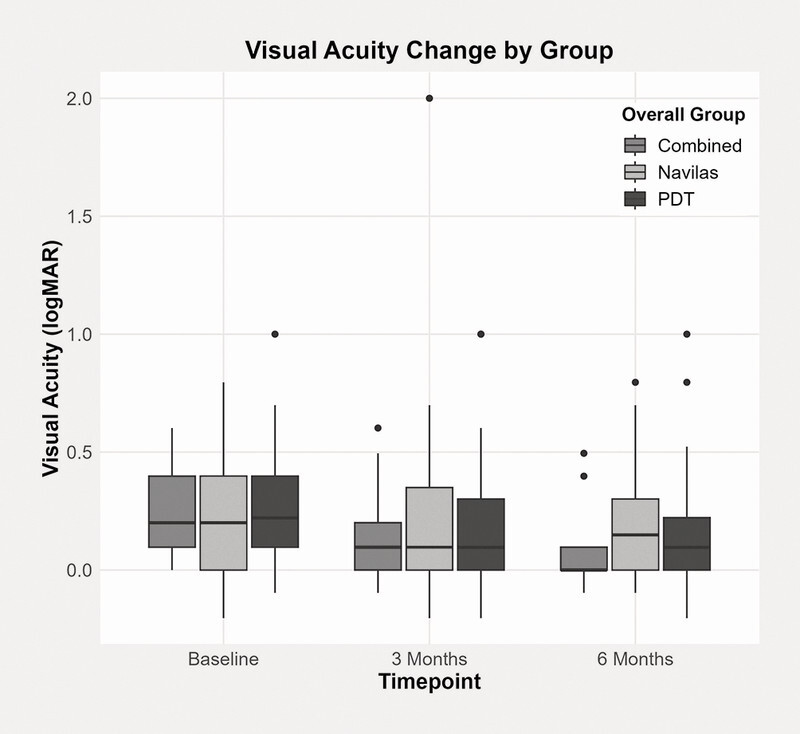
VA change by group.

There were no significant differences between the groups in terms of CST or VA at either three or six months ( Figs. 5 and 6 ). SRF was included in the central subfield thickness (CST) analysis if centrally located.

## Discussion

CSCR management remains a significant clinical challenge, which is mainly due to the complex and not fully understood pathophysiology of the condition, difficulties in precise classification, and limited effective treatment options 1 , 3 , 11 , 20 . In addition, the limited availability of verteporfin further restricts the use of PDT. In our study, we aimed to compare the efficacy of SML treatment and PDT treatment in resolving SRF and their impact on VA in patients with both acute and chronic CSCR.

Our findings indicate that both PDT and SML treatment led to statistically significant reduction in SRF and CST at both the three and six-month follow-up. In the “combined” group, significant improvements in both SRF and CST were observed only after six months. This suggests that this group is primarily composed of individuals with chronic cases, which is why clinicians had to consider both treatment options.

Surprisingly, we found no statistically significant differences between the PDT and SML groups in terms of SRF reduction and CST improvement, although previous studies such as the PLACE trial by Van Dijk et al. indicated PDTʼs superior efficacy 17 . This discrepancy may be due to differences in study design or cohort characteristics. In addition, the observed difference may be partially explained by the lack of randomisation of co-factors in our retrospective study. Although the two groups appear comparable, one group may have included a higher proportion of more advanced cases. Furthermore, the study by Van Dijk et al. included more than twice as many patients, which may have contributed to the observed differences 17 . It is also noteworthy that the incidence of chronic CSCR was considerably lower in the SML group than in the PDT and combined groups.

Despite SRF and CST reduction, our study did not reveal any statistically significant improvement in VA, which could be attributed to the rather small study cohort. Apart from that, the long median duration of subfoveal SRF before treatment (nine months for PDT, six months for SML, and fifteen months “combined”) may have limited the potential for functional improvements ( Table 1 ), as prolonged SRF presence may lead to irreversible changes in the RPE. Our cohort also had relatively good baseline visual acuity, which could lead to a smaller potential for improvement. This is consistent with the above mentioned large randomised prospective PLACE trial 17 . This also raises the question as to whether VA alone is a meaningful endpoint for CSCR patients, as VA may be less affected by SRF if the fovea is not directly involved. Nonetheless, even small, non-significant VA improvements could still hold clinical significance for individual patients.

One additional observation was a slight increase in SRF as well as CST in the PDT at six-month follow-up. This may be partly due to the exceptionally positive response seen at three months where median SRF was reduced to 0 µm. Alternatively, the six-month data may be skewed due to missing data from ten patients (30.30%), potentially introducing bias.

Limitations in this study include its retrospective study design potentially leading to bias and distortions, and confounding factors possibly causing a challenge in establishing causal relationships. Secondly, our patient cohort was relatively small, so generalisability is questionable. Thirdly, a potential inter-eye interaction must be considered in our analysis, as four out of eighty-one patients received bilateral treatment. Lastly, our data was incomplete with up to 30% of the data missing at the six-month follow-up visits compared to baseline. Apart from that, the study lacked a control group, thus precluding a comparison between the PDT and SML patients and a control or placebo group. Further research is required, which would of course include large, prospective, randomised controlled trials to evaluate our findings.

In conclusion, SML shows promise as a safe and accessible alternative while PDT appears to retain a slight efficacy advantage over SML, especially in SRF reduction. Given verteporfinʼs limited availability, SML presents a practical option in clinical settings, offering significant benefits in SRF reduction. SML is also widely available, significantly less costly, and carries a low risk of adverse effects, making it a valuable treatment option for CSCR patients.
